# Microaggressions: Prevalence and Perspectives of Residents and Fellows in Post-Graduate Medical Education in Kuwait

**DOI:** 10.3389/fsurg.2022.907544

**Published:** 2022-06-15

**Authors:** Asmaa Al Rashed, Rawan Al Yousef, Farah Alhouti

**Affiliations:** ^1^ Surgery Department, Amiri Hospital, Kuwait City, Kuwait; ^2^ Medical Student, University of Glasgow School of Medicine, Dentistry and Nursing, Glasgow, United Kingdom

**Keywords:** microaggressions, prevalence, residents, fellows, post-graduate medical education, Kuwait, surgery, training

## Abstract

**Objective:**

Microaggression prevalence in post-graduate medical education is unknown in Kuwait. The objective is to determine the prevalence of and capture the perspectives on microaggression among post-graduate trainees in Kuwait.

**Materials and Methods:**

This is a cross-sectional study of an anonymous online survey targeting residents and fellows in Kuwait. Data collected included demographics, level of training, country of training, microaggression experience, types, and response. Univariate and multivariate analyses were performed using the Chi-square test and binary logistic regression, respectively.

**Results:**

A total of 319 participants (69.1% females) included 52% junior residents, 39.2% senior residents, and 8.78% fellows. Forty-three percent were aware of the microaggression definition. The percentage was significantly higher in respondents from Gulf/Middle East Countries (57.9%) than from Kuwait. Approximately three-quarters experienced microaggressions. Senior residents were more likely to report exposure to microaggressions [Odds ratio (OR) = 2.4, *P* < 0.05] and had higher odds of exposure than juniors (OR = 9.85, *P* < 0.05). Exposure to microaggressions was highest in surgery/surgical specialties. The most common act of microaggression was verbal, followed by invalidation/dismissal of thoughts/ideas, and then acts of discrimination. Of those who experienced microaggressions, two-thirds thought that the experience had a psychological effect on them. Both groups reported low confidence in dealing with microaggressions (Gulf/Middle East Countries 18.8% and Kuwait 30.1%); the difference was not statistically significant.

**Conclusions:**

Microaggressions are common among post-graduate medical trainees in Kuwait. Implementation of strategies to manage it is necessary. Further research on its impact on medical-training outcomes is needed.

## Introduction

The term microaggression was first coined by American Harvard psychiatrist Chester Pierce to clarify the ambiguity surrounding commonplace non-verbal inequities inflicted by white Americans on African Americans in 1970 (1). Microaggressions are now used as an umbrella term for any derogatory verbal, behavioral, or visual insults directed towards a group of individuals (2, 3). Due to its disguised nature where both the offender and affected individual are usually unaware of the offence, the situation is often overlooked and devalued, leaving the group subject to microaggressions feeling confused and demoralized (2, 4, 5). Studies show that cumulative exposure to microaggressions can have detrimental effects, such as mental exhaustion, depression, hypertension, and suicidal thoughts (6–10). According to Sue et al., microaggressions can occur in the form of (1) micro-invalidations, which are actions of dismissal and invalidation of an individual’s feelings or thoughts; (2) micro-assaults, which show close overlap with overt racism and are considered as any derogatory behavior or words towards an individual; and (3) micro-insults, which is unconscious demeaning delivery of words and disregard of a person’s identity or heritage (2).

Microaggressions occur in medicine and healthcare as reported in the literature (11–20), and they have been mentioned as one of the factors that negatively impact the workplace environment and medical education in different regions of the world (16–18). There is expanding literature on the prevalence of microaggressions in the healthcare setting. Its occurrence is not exclusive to a specific profession and cases have been documented involving nurses (11, 12), surgeons (15, 21), physicians, medical students, and residents (14–19). Among healthcare professionals, microaggressions have been reported to cause depression, burnout, suicidal thoughts, and even lead to increased medical errors (11, 14, 15). The negative impact of microaggressions is also evident in medical education and can affect a student’s academic performance and well-being, as well as being a cause of increased workload and the feeling of not belonging also known as the imposter phenomenon (12, 17).

Unfortunately, there have been no published articles addressing microaggressions in medicine in Kuwait and the Arabian Gulf region to date. Recently, a study from Kuwait looked at the barriers preventing medical students and interns from choosing a surgical career and the solutions to those barriers and identified multiple deterrent factors such as long working hours, quality of life, and maternity and paternity leave policies. However, it also found in its thematic analysis that unprofessional attitudes, especially those of male surgeons, could render a surgery a harsh workplace (22, 23). Therefore, a closer examination of human interactions in the medical and healthcare workplaces in Kuwait and the Gulf region is needed, since demographic differences exist across the world and this can complicate the extrapolation of studies from regions that hold different social and cultural values. The objective of this study is to determine the prevalence of and capture the perspectives on microaggressions in post-graduate medical training programs in Kuwait, and to assess their prevalence in the Arabian Gulf countries considering the similarities in culture and demographics they share with Kuwait.

## Materials and Methods

A cross-sectional study was conducted through an online survey, using Google forms, containing 11 questions (see Appendix 1: Microaggression Questionnaire). Ethical approval was obtained from the Ministry of Health (MOH) and all participants consented anonymously. The data were collected between 21st and 24th of August 2020, and included information on demographics, level of training, country of training, experiences of microaggression, acts of microaggression, and responses to microaggression by the participants. The inclusion criteria were determined as the place of residency and fellowship training programs located in Kuwait, the Arabian Gulf region (Kingdom of Saudi Arabia, Kingdom of Bahrain, Qatar, Sultanate of Oman, United Arab Emirates), and the Middle East region (Yemen, Iraq, Palestine, Jordan, Lebanon, Syria). Participants who received post-graduate medical training in other countries were excluded. Statistical analysis was performed using R v 3.6.3. Counts and percentages were used to summarize the distribution of categorical variables. The Chi-square test of independence was used to assess the association between categorical variables. Multivariable analysis was performed using binary logistic regression and *post-hoc* analysis. Hypothesis testing was performed at a 5% level of significance.

## Results

### Demographics

The study sample included 319 respondents after exclusion (seven participants). At the time of the study, residency and fellowship programs in Kuwait Institute for Medical Specialization (KIMS) had a total of 801 trainees with 198 who responded, resulting in a response rate of 24.7% for participants from Kuwait. The gender of participants was 30.9% males and 69.1% females. Participants were from various specialties, such as surgery and surgical subspecialties (38.9%), medical (30.1%), dentistry (12.9%), pediatrics (6.27), and others (12%). About half of the respondents (52%) were junior residents and 39.2% were senior residents, while fellows were only 8.78%. Table 1A includes descriptive statistics of the study sample stratified by residency location.

**Table 1A:** Descriptive statistics for the study sample stratified by residency location.

	[All]	Gulf/Middle East regions	Kuwait	*P*
	*N = 319*	*N = 121*	*N = 198*	
Gender				0.016
Female	163 (69.1%)	69 (61.1%)	94 (76.4%)	
Male	73 (30.9%)	44 (38.9%)	29 (23.6%)	
Specialty				
Dentistry	41 (12.9%)	14 (11.6%)	27 (13.6%)	
Emergency medicine	1 (0.31%)	0 (0.00%)	1 (0.51%)	
Family medicine	6 (1.88%)	0 (0.00%)	6 (3.03%)	
Medical field	96 (30.1%)	22 (18.2%)	74 (37.4%)	
Neurology	1 (0.31%)	0 (0.00%)	1 (0.51%)	
Nuclear medicine	2 (0.63%)	0 (0.00%)	2 (1.01%)	
Other	28 (8.78%)	7 (5.79%)	21 (10.6%)	
Pediatrics	20 (6.27%)	6 (4.96%)	14 (7.07%)	
Surgery and surgical subspecialties (including OBGYN)	124 (38.9%)	72 (59.5%)	52 (26.3%)	
Current level of training				0.003
Junior resident (PGY1–PGY2)	166 (52.0%)	52 (43.0%)	114 (57.6%)	
Senior resident (PGY3–PGY5)	125 (39.2%)	51 (42.1%)	74 (37.4%)	
Fellow	28 (8.78%)	18 (14.9%)	10 (5.05%)	

### Prevalence and Experience of Microaggressions

Less than half of the respondents were aware of the term microaggressions (43.6%), and the percentage was significantly higher (*P* < 0.001) in Gulf and Middle East region respondents (57.9%) than respondents in Kuwait (34.8%). Approximately three-quarters of the respondents experienced microaggressions (71.5%). The percentage was significantly higher in respondents from the Gulf and Middle East respondents (79.3%) than in respondents from Kuwait (66.7%, *P* < 0.05). Regarding the response to microaggression, respondents from Gulf countries and the Middle East were more passive towards reporting microaggressions (63.5% vs. 54.5%). Overall, 15% discussed the matter with a senior in charge (15.4%), while 26.3% discussed the matter with the offender. Of those who experienced microaggressions, two-thirds thought that the experience had a psychological effect on them. When asked about feeling confident in dealing with microaggressions, there was no significant difference between both groups (*P* = 0.125), with 18.8% and 30.1% of respondents from the Gulf/Middle East and Kuwait, respectively, thinking that they had the confidence to deal with it. Table 1B includes descriptive statistics stratified by residency location.

**Table 1B:** Descriptive statistics for the study sample stratified by residency location.

	[All]	Gulf/Middle East regions	Kuwait	*P*
	*N = 319*	*N = 121*	*N = 198*	
Aware of the term microaggression				<0.001
No	180 (56.4%)	51 (42.1%)	129 (65.2%)	
Yes	139 (43.6%)	70 (57.9%)	69 (34.8%)	
Ever experienced microaggression				0.021
No	91 (28.5%)	25 (20.7%)	66 (33.3%)	
Yes	228 (71.5%)	96 (79.3%)	132 (66.7%)	
Response to microaggression				0.083
Discuss the matter with a senior in charge	35 (15.4%)	17 (17.7%)	18 (13.6%)	
Discuss the matter with the offender	60 (26.3%)	18 (18.8%)	42 (31.8%)	
I don’t usually do anything about it	133 (58.3%)	61 (63.5%)	72 (54.5%)	
Experience had a psychological effect				0.024
No	75 (33.0%)	23 (24.2%)	52 (39.4%)	
Yes	152 (67.0%)	72 (75.8%)	80 (60.6%)	
Confidence dealing with microaggressions				0.125
Maybe	81 (33.2%)	35 (34.7%)	46 (32.2%)	
No	101 (41.4%)	47 (46.5%)	54 (37.8%)	
Yes	62 (25.4%)	19 (18.8%)	43 (30.1%)	

### Microaggressions and Training Level

Analysis showed that senior residents were more likely to report exposure to microaggressions (OR = 2.4, *P* < 0.05) than junior residents. When the analysis was stratified by location, no significant association was observed between the training level and exposure to microaggressions among respondents in Kuwait (OR = 1.24, *P* > 0.05). Among residents in the Middle East/Kuwait, the odds of exposure were higher in senior residents than junior residents (OR = 9.85, *P* < 0.05), as shown in Table 2.

**Table 2:** Multivariate analysis of factors associated with exposure to Microaggressions.

Predictors	Overall			In Kuwait			Middle East/GCC		
	Odds ratios	CI	*P*	Odds ratios	CI	*P*	Odds ratios	CI	*P*
(Intercept)	2.45	1.19–5.33	0.018	1.67	1.00 –2.85	0.052	2.51	1.07–6.43	0.042
Gender: Male vs. female	0.91	0.47–1.80	0.786	1.57	0.61–4.44	0.365	0.55	0.20–1.50	0.239
Nationality: non-Kuwaiti vs. Kuwaiti	1.33	0.56–3.14	0.520	1.99	0.28–40.01	0.545	1.14	0.40–3.15	0.796
Location: in Kuwait vs. GCC and ME	0.66	0.30–1.41	0.292						
Training level: PGY1–PGY 2	Ref								
Training level: PGY3–PGY 5	2.40	1.23–4.94	**0**.**013**	1.24	0.54–2.93	0.612	9.85	2.54–65.65	**0**.**004**
Training level: Fellow	0.91	0.38–2.33	0.844	1.26	0.31–6.30	0.757	1.07	0.33–3.71	0.916

*Analysis was performed using binary logistic regression.*

### Acts of Microaggressions

There were no observed differences between those who were trained in Kuwait compared to those in the Gulf and ME regions regarding the act of microaggressions. The most common act of microaggression was a verbal insult (67.0%), followed by the invalidation of an opinion (62.1%), dismissal of thoughts and opinions (62.1%), and acts of discrimination (56.4%). The least common were passive-aggressive behavior (1.32%), followed by gender discrimination (0.44%), and loss of learning opportunities (0.44%). Table 3 shows the incidence of microaggressions stratified by location.

**Table 3:** Incidence of microaggressions stratified by location.

Microaggression act	[All]	Gulf/Middle East region	Kuwait	*P* overall
	*N = 319*	*N = 121*	*N = 198*	
Acts of discrimination	128 (56.4%)	57 (59.4%)	71 (54.2%)	0.522
Invalidation of an opinion	141 (62.1%)	69 (71.9%)	72 (55.0%)	0.014
Dismissal of thoughts and opinions	141 (62.1%)	69 (71.9%)	72 (55.0%)	0.026
Verbal insult	152 (67.0%)	71 (74.0%)	81 (61.8%)	0.076
Loss of learning opportunities	1 (0.44%)	1 (1.04%)	0 (0.00%)	0.423
Gender discrimination	1 (0.44%)	1 (1.04%)	0 (0.00%)	0.423
Passive aggressive behavior	3 (1.32%)	3 (3.12%)	0 (0.00%)	0.074

### Microaggressions and Specialty

**Figure 1:**
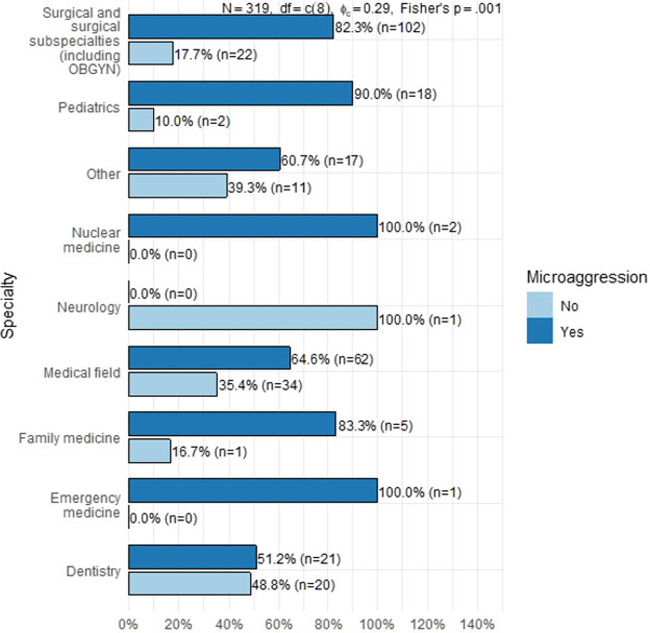
Exposure to microaggressions by specialty.

Figure 1 demonstrates exposure to microaggressions according to specialty. As noted, the highest number of microaggressions reported by specialty were in surgery and surgical subspecialties followed by medicine and then dentistry. It is worth mentioning that the numbers of participants from other specialties were very low to consider accurate representation and comparison.

A pairwise comparison of specialties (see Table 4) showed that the odds of exposure to microaggression were 80% lower in dentistry residents than those in family medicine residents (OR = 0.21, *P* = 0.75), although the association was not statistically significant at the 0.05 level. The odds of reporting exposure to microaggression were ∼90% lower in dentistry residents than surgery residents (OR = 0.222, *P* = 0.0016). The odds of reporting exposure to microaggressions were also lower in medical field residents than in surgery residents (OR = 0.386, *P* = 0.032). None of the remaining pairwise comparisons were statistically significant at the 0.05 level. The odds of exposure to microaggression were higher in pediatric residents than in dentistry residents (OR = 0.117, *P* = 0.08), although the association was only significant at the 0.1 level.

**Table 4:** Pairwise comparisons of specialties.

	Dentistry	Family medicine	Medical field	Other	Pediatrics	Surgery and surgical subspecialties (including OBGYN)
Dentistry	**0** **.** **512**	0.7451	0.6906	0.9714	0.0838	**0**.**0016**
Family medicine	0.21	**0**.**833**	0.9457	0.9146	0.9978	1
Medical field	0.576	2.742	**0**.**646**	0.999	0.3089	**0**.**032**
Other	0.679	3.235	1.18	**0**.**607**	0.2881	0.1329
Pediatrics	0.117	0.556	0.203	0.172	**0**.**9**	0.9632
Surgery and surgical subspecialties (including OBGYN)	0.222	1.058	0.386	0.327	1.904	**0**.**825**

*Diagonals represent the probability of exposure to microaggression. The upper triangle represents the P-values for post-hoc comparisons. The lower triangle represents the odds ratio (column/row). Analysis was performed using logistic regression followed by post-hoc comparisons of estimated marginal probabilities.*

## Discussion

This is the first study to address microaggressions in post-graduate medical education in Kuwait. The most important finding was the high prevalence of microaggressions that was reported with a rate of 71.5% (Kuwait 66.7%, the Gulf and ME 79.3%) among all participants from post-graduate residency and fellowship programs. Interestingly, more than half of the participants (56.4%) were not aware of the term “microaggressions” at the time they participated in this study, with the lowest awareness among Kuwait’s post-graduate trainees comparable to the Gulf/ME (65.2% vs 42.1% *P* < 0.001), which was statistically significant. This indicates the importance of raising awareness about microaggressions and warrants further study to outline interventions and solutions that could decrease the likelihood of microaggressions in medical education in Kuwait and in the Middle East region.

To outline a few limitations of this study, the questionnaire was short and did not address details about the psychological effect and response to microaggressions; however, the goal was to capture a general perspective since the topic is new and the study was conducted over a short period of time (only 3 days).

Although most participants were females (69.1%), which is consistent with the predominance of female medical school graduates in Kuwait and the Gulf region (23). However, there was no difference related to gender in terms of experiencing microaggressions (*odds ratios* 0.91 [CI 0.47–1.80], *P* =0.786). Although gender bias has been documented in the literature (24, 25), being of a minority group regardless of gender was the most common factor for being subjected to microaggressions as reported by a study from North America (26).

Contrary to what is reported in the literature in a study from Iran and a study from the United States (16, 24), that junior residents are more likely than senior residents or attending surgeons to experience microaggression, in our study, senior residents were more likely to be exposed to microaggressions (OR = 9.85, *P* < 0.05) and report exposure to microaggressions (OR = 2.4, *P* < 0.05) than junior residents, regardless of the country of the training program. This could suggest that as residents advance into their training, changes in human interactions and behavior when faced with microaggression may occur. However, the study did not include staff and faculty nor did it identify perpetrators and therefore this should be taken into consideration.

While no differences were observed regarding the acts of microaggression based on the location of training, it was noted that the most common act of microaggressions was the verbal microaggression followed by the invalidation of an opinion, dismissal of thoughts and opinions and then acts of discrimination. Another limitation of this study is that microaggression is influenced by what is deemed socially acceptable. For example, calling a physician by their first name and using the title rather than their last name in the medical field is considered acceptable in the ME (27). However, some may consider this as an act of microaggression by dismissing the person’s qualifications. Furthermore, since this study has not explored the exact scenarios of the reported microaggressions, some of the reported microaggressions may also fall into the overt acts of discrimination (macroaggression) category rather than microaggression.

While findings of high rates of microaggressions by specialty were found in surgery and surgical subspecialties (82.3%), followed by medical (30.1%) and dentistry (12.9%), these findings were consistent with reports from regions outside the Gulf/ME region. Microaggression and implicit bias in surgical training has been reported, in an American study by Alimi Yewande et al., in a national survey with a majority (72.2%, *n* = 1173) of respondents reported experiencing microaggressions, most commonly from patients (64.1%), followed by staff (57.5%), faculty (45.3%), and co-residents (38.8%), while only a small proportion (*n* = 109, 7.0%) of residents reported these events to the graduate medical education office/program director, and nearly one-third (30.8%) of residents said they experienced retaliation after reporting a microaggression (20).

The negative impact of microaggression has been documented by many researchers (11, 12, 14, 15, 19). This paper reflects these studies, as two-thirds of those who experienced microaggression thought that these incidents had a psychological effect on them. However, further studies are needed to investigate the characteristics of these psychological effects, and the impact they have on the quality of post-graduate medical education in Kuwait and the ME region.

To conclude, microaggressions are prevalent and common in post-graduate medical education in Kuwait, the Gulf countries, and the Middle East. Implementation of strategies to raise awareness of and to manage them is necessary, along with further research on their impact on medical-training outcomes.

## Data Availability

The raw data supporting the conclusions of this article will be made available by the authors, without undue reservation.
