# Supplementary material for: Microaggressions: Prevalence and Perspectives of Residents and Fellows in Post-Graduate Medical Education in Kuwait
Source: Front Surg. 2022 Jun 15;9:907544. doi: 10.3389/fsurg.2022.907544 (PMC9302556; doi:10.3389/fsurg.2022.907544)
Supplement: Supplementary file 1 [file Data_Sheet_1_v1.pdf]

	Microaggression Questionnaire
1	I agree to voluntarily participate in this survey, which will be used for research purposes.<br>▪ Yes<br>▪ No
2	Gender<br>▪ Male<br>▪ Female
3	What is your specialty?<br>▪ Surgery and surgical subspecialties (including OBGYN)<br>▪ Pediatrics<br>▪ Dentistry<br>▪ Medical field<br>▪ Neurology<br>▪ Nuclear medicine<br>▪ Family medicine<br>▪ Other
4	What is your current level of training?<br>▪ Junior resident (PGY1-PGY2)<br>▪ Senior resident (PGY3-PGY5)<br>▪ Fellow
5	What is your nationality? (free text)
6	Where are you currently working?<br>▪ Resident abroad<br>▪ Resident in Kuwait
7	Are you aware of the term 'microaggression'?<br>▪ Yes<br>▪ No
8	"Microaggression (Merriam-Webster): a comment or action that subtly and often unconsciously or unintentionally expresses a prejudiced attitude toward a member of a marginalized group (such as a racial & ethnical minority, gender-based, etc.)."

	Have you ever experienced microaggression before? If not, thank you for your participation. You may end the survey here.<br>▪ Yes<br>▪ No
9	What kind of incident have you been through? (Select all answers applicable)<br>▪ Verbal insult<br>▪ Physical insult<br>▪ Loss of learning opportunities<br>▪ Acts of discrimination<br>▪ Gender discrimination<br>▪ Passive aggressive behavior<br>▪ Invalidation of an opinion
10	How do you normally respond to microaggression?<br>▪ I don't usually do anything about it<br>▪ Discuss the matter with a senior in charge<br>▪ Discuss the matter with the offender
11	Has this experience affected you in any way? (i.e. psychologically, physically, etc.)<br>▪ Yes<br>▪ No
12	Do you feel confident dealing with microaggressions in the workplace?<br>▪ Yes<br>▪ Maybe<br>▪ No
